# Physiological Response of Olive Trees Under *Xylella fastidiosa* Infection and Thymol Therapy Monitored Through Advanced IoT Sensors

**DOI:** 10.3390/plants14091380

**Published:** 2025-05-02

**Authors:** Claudia Cagnarini, Paolo De Angelis, Dario Liberati, Riccardo Valentini, Valentina Falanga, Franco Valentini, Crescenza Dongiovanni, Mauro Carrieri, Maria Vincenza Chiriacò

**Affiliations:** 1 Istituto per la Protezione e la Ricerca Ambientale (ISPRA), 00144 Rome, RM, Italy; 2 CMCC Foundation—Euro-Mediterranean Center on Climate Change, 01100 Viterbo, VT, Italy; rik@unitus.it (R.V.); mariavincenza.chiriaco@cmcc.it (M.V.C.); 3 Dipartimento per la Innovazione nei Sistemi Biologici, Agroalimentari e Forestali, Università degli Studi della Tuscia, 01100 Viterbo, VT, Italy; pda@unitus.it (P.D.A.); darioliberati@unitus.it (D.L.); 4 Dipartimento di Bioscienze e Territorio, Università degli Studi del Molise, 86090 Pesche, IS, Italy; v.falanga@studenti.unimol.it; 5 CIHEAM Bari, International Center for Advanced Mediterranean Agronomic Studies, 70010 Valenzano, BA, Italy; valentini@iamb.it; 6 Centro di Ricerca, Formazione e Sperimentazione in Agricoltura “Basile Caramia” (CRSFA), 70010 Locorotondo, BA, Italy; enzadongiovanni@crsfa.it (C.D.); maurocarrieri@crsfa.it (M.C.)

**Keywords:** spectral transmittance, sap velocity, TreeTalkers, proximity sensors, nanoparticles, bacterial plant diseases, active natural molecules, *Xfp* epidemic, Apulia region

## Abstract

Since its first detection in 2013, *Xylella fastidiosa* subsp. *pauca* (*Xfp*) has caused a devastating Olive Quick Decline Syndrome (OQDS) outbreak in Southern Italy. Effective disease surveillance and treatment strategies are urgently needed to mitigate its impact. This study investigates the short-term (1.5 years) effects of thymol-based treatments on infected olive trees of the susceptible cultivar Cellina di Nardò in two orchards in Salento, Apulia region. Twenty trees per trial received a 3% thymol solution either alone or encapsulated in a cellulose nanoparticle carrier. Over two years, sap flux density and canopy-transmitted solar radiation were monitored using TreeTalker sensors, and spectral greenness indices were calculated. *Xfp* cell concentrations in plant tissues were quantified via qPCR. Neither thymol treatment halted disease progression nor significantly reduced bacterial load, though the *Xfp* cell concentration reduction increased over time in the preventive trial. Symptomatic trees exhibited increased sap flux density, though the treatment mitigated this effect in the curative trial. Greenness indices remained lower in infected trees, but the response to symptom severity was delayed. These findings underscore the need for longer-term studies, investigation of synergistic effects with other phytocompounds, and integration of real-time sensor data into adaptive disease management protocols.

## 1. Introduction

The plant pathogenic bacterium *Xylella fastidiosa* subsp. *pauca* (*Xfp*), previously known to affect various host plants in the Americas, was identified in Salento, the southernmost province of the Apulia region, Italy, in 2013, where it was associated with Olive Quick Decline Syndrome (OQDS) [1]. The traditional olive (*Olea europaea* L.) cultivars Ogliarola Salentina and Cellina di Nardò, which are widely represented in the area, turned out to be susceptible to *Xfp* infection, as they harbor high bacterial cell concentrations and develop severe symptoms [2]. Symptoms can manifest some months after infection and evolve from leaf scorching and scattered desiccation of twigs and branches [3]. Dead leaves remain attached to the twigs throughout summer and begin to drop with the first rains in autumn. Initially, symptomatic leaves and twigs are localized at the top of the crown; then, they expand to the rest of the canopy and can culminate in plant death in as fast as 2–5 years [3]. The symptoms caused by *Xfp* are highly variable, depending on different factors, and are nonspecific, as they appear like those caused by water stress [4,5]. The *Xfp* bacterium is transmitted to new host plants by sap-feeding insect vectors. In Europe, the most abundant and widely spread xylem sap-feeder insect is the spittlebug *Philaenus spumarius* L., which is therefore considered the key vector of the *Xfp* epidemic in Apulia [6].

Controlling *Xfp* is very difficult because of its localization in the xylem and the high virulence of the *Xfp* strain. Since the first identification of *Xfp* in Apulia’s olive orchards, numerous substances (e.g., synthetic chemicals, natural products, biological agents) have been tested in vitro and in planta to control the pathogen [7]. In mid-term experimental field trials carried out on olive orchards in Salento, the application of a biocomplex based on copper, zinc, and citric acid in combination with suitable agronomic practices significantly reduced both the symptoms (twigs and branch wilting) and bacterial cell density [8]. Several trials verified the effects of N-acetylcysteine, which had previously shown promising results in sweet orange plants [9], against rapid desiccation syndrome in olive trees [10]. In treated plants, a slowdown in the progression of the disease was observed when the product was injected; however, in the long term, no significant differences were observed in the ability to attenuate the symptoms or reduce the bacterial load. The authors of [11] tested a natural bioactive detergent in a spray based on vegetable oils and extracts from different botanical species. They showed a reduction in the severity of both the disease and bacterial load as well as an induced activation of the defenses of the plants; however, their observations referred to a limited period and number of plants. Promising results in reducing *Xfp* symptom severity were also obtained in long-term experiments [12] by treating with a hydroalcoholic extract from pomegranate. These findings collectively highlight the potential of various natural products and synthetic chemical treatments in mitigating *Xfp* symptoms, yet they also underscore the need for further exploration of more effective and scalable alternatives, such as thymol, which proved to have antimicrobial and antibiofilm properties [13,14].

Soon after the first detection of *Xfp* in Apulia, the Regional Phytosanitary Services established demarcated areas that are constantly updated according to European legislation. In the containment and buffer zones, plant infection status is periodically monitored to detect new outbreaks and contrast the spread of the disease. Several molecular techniques were developed, and protocols were optimized to detect infected olive trees before symptom appearance [15]. The use of aerial high-resolution imagery for pre-visual detection of infected trees was recently demonstrated [16], since *Xfp* affects plant traits at both the canopy level (structure) and leaf level (pigments), thus causing distinctive changes in the reflectance and radiance spectra [16]. Broadband high-spatial-resolution satellite imagery was successfully used to monitor olive grove restoration from *Xfp* infection at both the field and tree levels [17]. While having lower spectral and spatial information, satellite sensors have the advantage of being inexpensive and not facing any endurance or regulatory limitations like aerial platforms. However, cheaper and easily deployable technological solutions capable of continuously monitoring *Xfp* infection at early stages would be highly valuable in containing the epidemic. Among existing technologies, TreeTalkers offer a promising and cost-effective alternative to aerial or satellite remote sensing, as they can be permanently installed on individual trees to collect high-frequency physiological and environmental data. Unlike aerial platforms or satellite sensors, TreeTalkers are not subject to regulatory constraints, weather limitations, or infrequent acquisition intervals, making them suitable for long-term, real-time monitoring at the tree scale. Their scalability and ease of deployment across large scales make them an attractive option for early detection and precision management of *Xfp*-infected olive groves [18,19].

Olive cultivars Leccino, FS17, Leccio del Corno, and Lecciana are resistant to *Xfp* because of their low symptom expression and bacterial cell concentration [20,21]. While the co-occurring mechanisms conferring resistance and susceptibility have not been fully elucidated, recent efforts have focused on the plant vascular system. It is generally agreed that water transport is affected by the occlusion of the xylem vessels by microbial aggregates, tyloses, and gels produced by the plant to isolate the bacterium [22,23]. Such xylem vasculature compartmentalization, which reduces hydraulic conductivity, is strong in susceptible cultivars. Susceptible cultivars are also more vulnerable to embolism by having fewer and larger xylem vessels [24,25,26] and undergoing more severe degradation of the pit membranes [23], which is induced by the bacterium to spread between adjacent vessels of the host plant [22]. Susceptible cultivars reported altered stomatal conductance and stem water potential upon infection [27]. The authors of [27] suggested that screening of the olive genotypes for resistance to *Xfp* could be accomplished by measuring the plant’s physiological response to infection. Since the type, strength, and timing of the plant’s defense mechanisms are cultivar-specific and play a major role in shaping the *Xfp* susceptibility [25,28], understanding these responses is also crucial for informing therapeutic strategies, such as the use of thymol, which has shown potential in reducing xylem occlusion by disrupting bacterial biofilms, thus preserving vascular functionality and reducing disease severity [13,14].

In this study, TreeTalkers were instrumented on n. 40 trees of two olive groves (n. 20 trees in Mesagne and n. 20 trees in Avetrana) in the epidemic areas of progression of the *Xfp* disease in Salento, Apulia region (Southern Italy), planted with the susceptible cultivar Cellina di Nardò. Two-thirds of the trees were treated with a natural substance based on thymol extract in spray, alone (treatment *thym*) or encapsulated in cellulose nanoparticles (treatment *thym + nano*), while one-third was left untreated (*ctr).* The first objective was to test the preventive and curative effects of the short-term (1.5 years) thymol application. The second objective was to investigate the response of the sap flux density in the xylem (or sap velocity *J*) to the *Xfp* infection and therapy application by continuous monitoring with TreeTalkers. The third objective was to test the capability of TreeTalkers to monitor the *Xfp* infection, OQDS disease progression, and therapy application by using a transmittance-based Normalized Difference Vegetation Index (*tNDVI*). The *tNDVI* is an analog to the well-known reflectance-based *NDVI* that exploits the radiation transmitted through the canopy and thus is not affected by the understory.

## 2. Results

### 2.1. Xfp Disease Patterns

**Figure 1:**
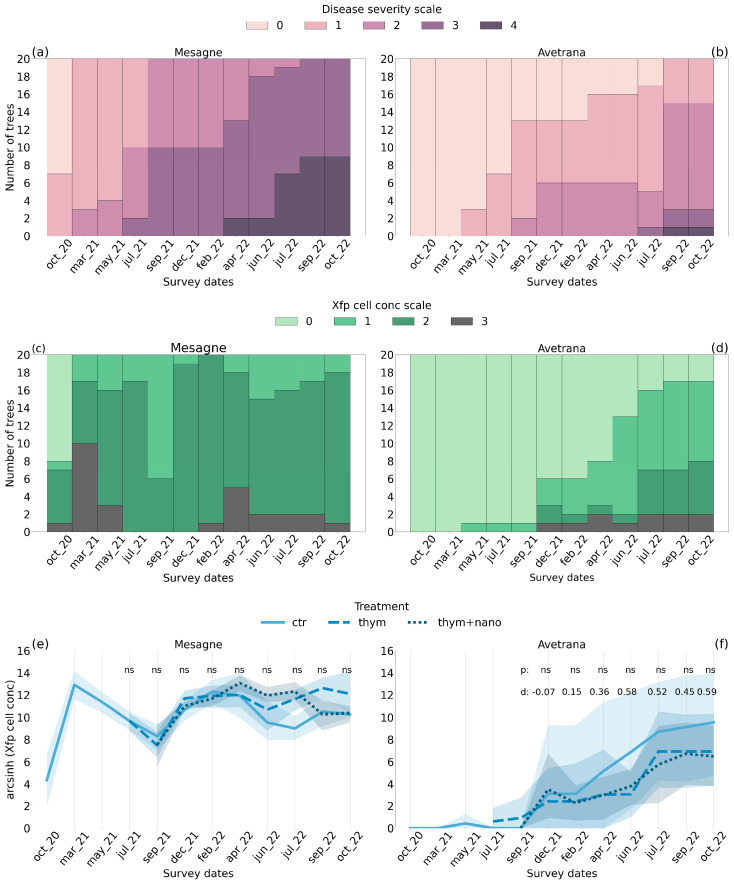
The number of trees belonging to the different classes of disease severity and *Xfp* cell concentration, respectively, (**a**–**c**) in Mesagne and (**b**–**d**) Avetrana (**b**–**d**) at the survey dates. Mean and 95% confidence interval of the *Xfp* cell concentration after *arcsinh* transformation, respectively, (**e**) in Mesagne and (**f**) Avetrana at the survey dates under *ctr*, *thym*, *thym + nano* treatments. ns: non-significant (Kruskal–Wallis *p* > 0.1). Cohen’s d between *ctr* and *thym + nano* at the survey dates is presented in (**f**).

*Xfp* symptom severity, assessed by expert technicians and scored on a scale of five levels, worsened progressively in both olive groves during the trials. Progression was remarkably rapid in the curative trial of Mesagne, where the disease severity started in classes 0–2 in October 2020 and ended in classes 3–4 in October 2022 (Figure 1a). Therefore, at the end of the trial, most of the twenty trees in Mesagne were highly damaged and displayed *Xfp* symptoms in 75–100% of the canopy. In the preventive trial of Avetrana, the first symptoms appeared only in May 2021 (Figure 1b). After building up during the summer of 2021, the disease severity plateaued until July 2022, when it started increasing again. At the end of the trial in October 2022, most of the twenty trees were mildly symptomatic between classes 1 and 2, with a few individuals displaying more severe symptoms in classes 3 and 4.

In Mesagne, the *Xfp* cell concentration, quantified via qPCR from plant material collected from the four quadrants of the crown and scored on a scale of four levels, displayed seasonal variability, characterized by spring peaks and summer declines (Figure 1c). At the beginning of the trial, more than half of the trees were healthy. After a steep increase in March 2021, all the trees were infected with medium-high loads between classes 2–3. Starting from May 2021, the *Xfp* cell concentration decreased to a minimum in September 2021: the mean *Xfp* cell concentration of the site in September 2021 was 97% lower than in May 2021. In the winter of 2021 and spring of 2022, the *Xfp* load increased smoothly and plateaued in the summer of 2022. In Avetrana, only one tree became infected from May 2021 to September 2021 (Figure 1d). From December 2021, the *Xfp* cell concentration increased, with a plateau in the summer of 2022. At the end of the trial, a few healthy individuals coexisted with a prevalence of low-medium infection trees.

The thymol-based treatments, first applied in May 2021 until October 2022, did not reduce the symptom severity. In Mesagne, the treatments did not affect the *Xfp* cell concentration (Figure 1e), while in Avetrana, *thym* and *thym + nano* tended to alleviate the bacterial load, ending in October 2022 with mean *arcsinh* values of 9.6 for *ctr* and 6.5 for both *thym* and *thym + nano* (Figure 1f). The treatment effect on *Xfp* cell concentration in Avetrana was not significant at any survey date according to the non-parametric Kruskal–Wallis test. However, the effect size on the bacterial load between *ctr* and *thym + nano* calculated with Cohen’s d showed a progressive increase in Avetrana starting from April 2022, reaching moderate values in the summer and autumn of 2022 (Figure 1f). The effect size of the bacterial load between *ctr* and *thym* was similar to *thym + nano*, but with smaller Cohen’s d values (21 July: −0.31, 21 September: −0.51, 21 December: 0.12, 22 February: 0.12, 22 April: 0.31, 22 June: 0.71, 22 July: 0.31, 22 September: 0.39, 22 October: 0.46). The results indicate that the overall effect of the thymol-based treatments on *Xfp* cell concentration reduction in Avetrana was modest but tended to increase over time, particularly for *thym + nano*, suggesting a possible cumulative effect.

### 2.2. Climate and Tree Physiology Time Trends

**Figure 2:**
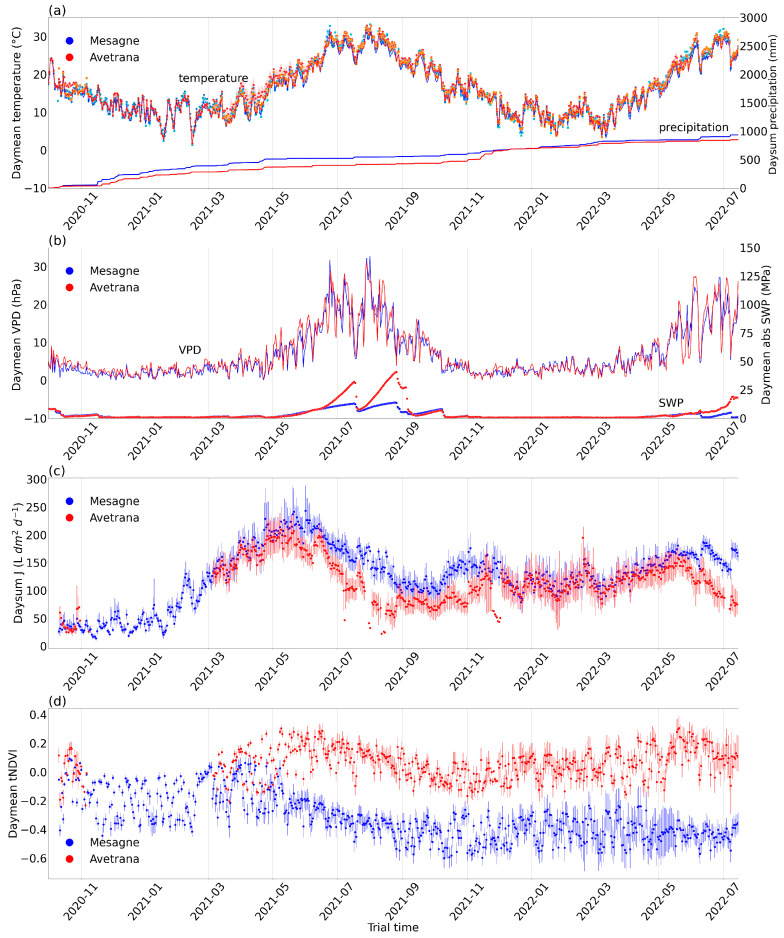
Temporal patterns in Mesagne and Avetrana of (**a**) daymean 2 m air temperature from the meteo stations (−), daymean under-canopy temperature from TreeTalkers (■), daymean sapwood temperature from TreeTalkers (●), and daysum precipitation from the meteo stations (right axis); (**b**) daymean vapor-pressure deficit *VPD* from the meteo stations and daymean (absolute) soil water potential (*SWP*) from TTsoils (right axis), (**c**) daysum sap flux density *(J*) from TreeTalkers, and (**d**) daymean transmittance-based *NDVI* (*tNDVI*) from TreeTalkers. *tNDVI* site intercomparison is not possible due to different scaling windows. TreeTalker’s data are shown with the mean and 95% confidence interval.

During the trials, the under-canopy air temperature and tree sapwood temperature, measured by TreeTalkers implanted in the trunk, one per tree, were close to each other and slightly higher than the 2 m air temperature of the meteo stations, although they were well aligned (Figure 2a). The sapwood temperature, recorded by the TreeTalkers’ needles implanted in the trunk, is likely closer to the values perceived by the *Xfp* cells in the xylem. The summer of 2021 was extremely hot, with a maximum air temperature of 41 °C registered by the meteo stations in the two sites and a maximum sapwood temperature of 44.1 °C and 43.1 °C in Mesagne and Avetrana, respectively. The summer of 2022 was less extreme, with a maximum air temperature of 38.2 °C and maximum sapwood temperatures of 39.4 °C and 40.3 °C in Mesagne and Avetrana, respectively. A marked decrease in *Xfp* cell concentration was recorded in Mesagne in the summer of 2021 (Figure 2c) when very high temperatures occurred in the sapwood of the olive trees (Figure 2a). In the summer of 2022, when the temperatures were less extreme (Figure 2a), *Xfp* cell concentration remained substantially unchanged in the two sites (Figure 2c,d).

In Mesagne, the first year of the experiment, from the 1st of October 2020 to the end of September 2021, registered a total precipitation of 564 mm/y, which was close to the long-term mean for the sites, whereas Avetrana was drier with a total precipitation of 425 mm/y (Figure 2a). The opposite occurred in the second year, from the 1st of October 2021 to the end of September 2022, with a total precipitation of 436 mm/y in Mesagne and 515 mm/y in Avetrana (Figure 2a). In the summer of 2021, a maximum vapor-pressure deficit (*VPD*) of 66 hPa was reached in both Mesagne and Avetrana, while it was 55 hPa in the summer of 2022 (Figure 2b). Two intense heatwaves, characterized by high temperature and *VPD* values, occurred at the end of June and July 2021. Another less severe heatwave occurred at the end of June 2022. After each heatwave, the soil water potential (*SWP*) measured by TTsoils rapidly increased in absolute value.

At the two sites, the sap flux density (*J*), measured according to the thermal dissipation method, peaked first in May 2021, while the second peak in May 2022 was far less pronounced, likely due to damage accumulation (Figure 2c). During the summers, *J* was higher in Mesagne, the site most severely affected by the disease, than in Avetrana. The small peak in November 2021, primarily visible in Mesagne, could be attributed to a late resprouting observed in the field as soon as the soil water was replenished. *J* in Mesagne was more responsive to climatic conditions than in Avetrana with higher correlation to air temperature, solar radiation, and *VPD* (Supplementary Figure S1); the correlation with *SWP* was of opposite sign between the two sites (Supplementary Figure S1). *J* was also responsive to single climatic events in both sites: in the hot and dry summer of 2021, *J* declined steadily, as expected due to the progressive reduction in the soil available water and tighter stomatal control, which peaked after significant rainfall events and suddenly dropped during heatwaves (Supplementary Figure S2).

The transmittance-based *NDVI (tNDVI)* patterns measured by TreeTalkers resembled the expected reflectance-based *NDVI* dynamics and were interpreted accordingly (Figure 2d). Due to the different scaling windows used for sensor intercompatibility, the tNDVI cannot be compared between the sites. The *tNDVI* did not show any strong seasonal variations, as anticipated for evergreen trees. Starting from May 2021, the *tNDVI* in Mesagne initiated a decreasing trend, thus capturing the progressive damage of the canopy. In Avetrana the first greenness peak occurred in May 2021, thus in phase with the sap flux density peak. The *tNDVI* in Avetrana peaked again in May 2022, while it did not in Mesagne.

### 2.3. Sap Flux Density

**Figure 3:**
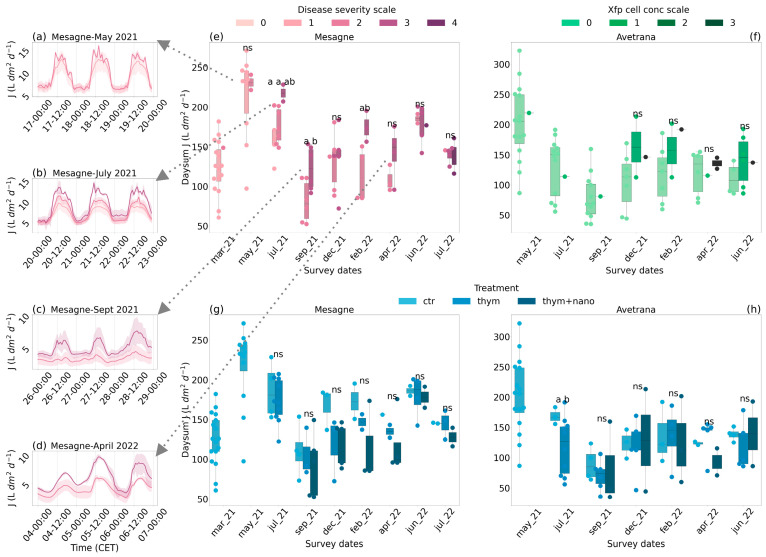
Hourly sap flux density (*J*) grouped by symptom severity classes in Mesagne during 3-day windows centered on the survey dates of (**a**) May 2021, (**b**) July 2021, (**c**) September 2021, and (**d**) April 2022. Daysum *J* grouped by (**e**) symptom severity classes in Mesagne, (**f**) *Xfp* cell concentration classes in Avetrana, (**g**) applied treatments in Mesagne, and (**h**) Avetrana, with dots representing individual measured values and boxplots displaying statistics (median, 25 and 75 percentiles). For readability, the *y*-axis scale is different between Mesagne and Avetrana. ns: non-significant (Kruskal–Wallis *p* > 0.1); a and b indicate different groups (Dunn adjusted *p* < 0.1).

The *Xfp* disease tended to increase *J*, though differently between the two sites. The high data uncertainty and uneven sample size distribution between the disease classes confounded the significance of the non-parametric Kruskal–Wallis test. In the curative trial of Mesagne, the trees in the symptom severity class 3 had significantly higher *J* than trees in class 2 (Figure 3e). Exceptions occurred in June 2022 and July 2022 when *J* did not visually differ between classes 2, 3, and 4, and at the beginning of the trial when *J* was similar between classes 1 and 2. Over the trial, the mean increase in *J* between adjacent symptom classes was 30%. In Mesagne, the *Xfp* cell concentration classes did not correspond to different *J* values, likely due to the fluctuating trend of the bacterial load in this site. For Mesagne, hourly *J* values during three consecutive days centered on the survey dates and differentiated by symptom severity class are shown in Figure 3a–d. In May 2021, the class 2 trees started displaying small peaks in the light, suggesting an open–close control of the leaf stomata (Figure 3a). In July 2021, the more severe the symptoms, the higher and more fragmented *J* in the light, with a clear distinction between the symptom classes (Figure 3b). In September 2021 and April 2022, the class 3 trees showed higher *J* than the class 2 trees both in the light and dark (Figure 3c,d).

In Avetrana, disease severity did not consistently affect *J,* likely because until 2022 the visual symptoms were not caused by the *Xfp* infection. Trees harboring higher *Xfp* cell concentration showed higher *J* (Figure 3f), particularly at the early phase of infection between the healthy trees and the class 1 trees. However, the effect was never statistically significant, mostly due to the low sample size of the infection classes. Over the whole trial, the mean increase between adjacent *Xfp* cell concentration classes was 30%.

In Mesagne, from December 2021, olive trees receiving the thymol-based treatments showed lower *J* values than *ctr* trees by −15% on average, although the effect was never significant due to uneven sample size distribution between classes (Figure 3g). The treatment *thym + nano* appeared more effective in lowering *J* than *thym* by −9% on average from December 2021. The treatment effect was opposite to that caused by the *Xfp* disease. In Avetrana, the decreasing effect of the applied treatments on *J* was visible at a few survey dates and was significant only in July 2021 (*p* = 0.063) (Figure 3h).

### 2.4. Leaf Traits

The leaf biochemical and morphological traits were characterized in four olive trees per site at the beginning of June 2021. In June 2021, the symptom severity in Mesagne was moderate, with most trees belonging to class 2 and the rest to class 3, while the *Xfp* cell concentration was high, with most individuals belonging to class 2 and a few already to class 3 (Figure 1a,c). In June 2021, in Avetrana, only one tree was infected by *Xfp* (Figure 1d). Leaves in Mesagne had a similar *Vc_max_* to Avetrana (Table 1) but a significantly higher *J_max_* (mean values of 198 and 156 µmol m^−2^ s^−1^ in Mesagne and Avetrana, respectively). The *LMA* was slightly, but not significantly, higher in Mesagne than in Avetran (Table 1). Leaf nitrogen content expressed by leaf area units (*N_area_*) did not differ between the two sites (Table 1) and was linearly correlated to *J_max_* (R^2^ = 0.48).

The stomatal conductance measured at field conditions before noon (*gsw* mean values of 0.16 and 0.10 mol m^−2^ s^−1^ in Mesagne and Avetrana, respectively) and transpiration before noon (*E* mean values of 3.26 and 2.62 mmol m^−2^ s^−1^ in Mesagne and Avetrana, respectively) were significantly higher in Mesagne than in Avetrana (Table 2). After noon, the transpiration rate and stomatal conductance in Mesagne decreased and aligned with the values in Avetrana (Table 2). Transpiration measured under field conditions at different times of the day agreed with the hourly patterns of *J*, showing stomatal opening until the early afternoon (Supplementary Figure S3).

### 2.5. Canopy Transmitted Radiation

**Figure 4:**
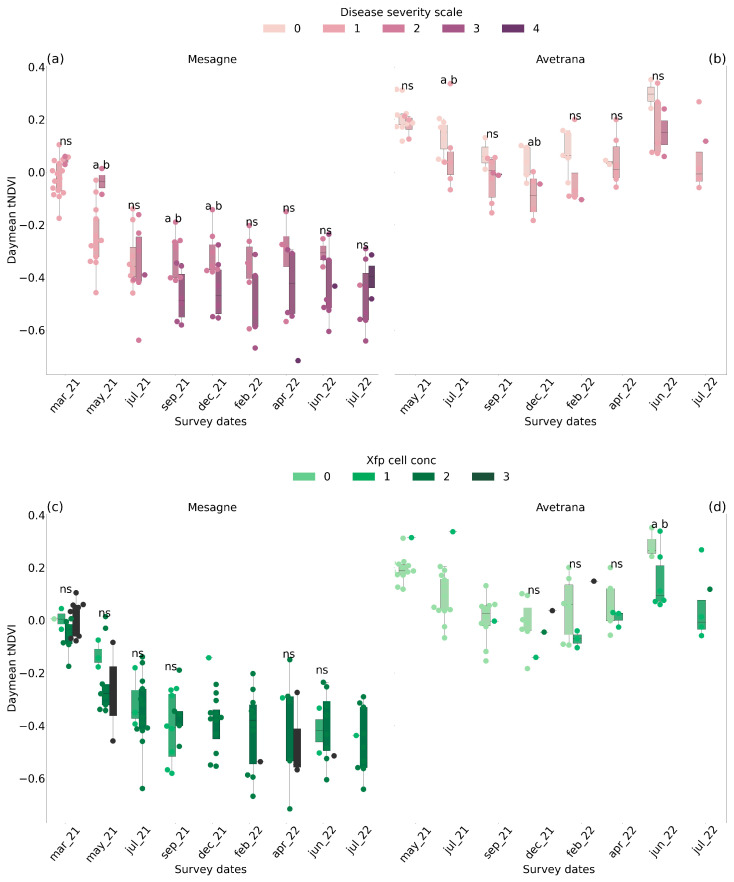
Daymean *tNDVI* at survey dates grouped by symptom severity (**a**) in Mesagne and (**b**) in Avetrana, *Xfp* cell concentration (**c**) in Mesagne and (**d**) in Avetrana, with dots representing individual measured values and boxplots displaying statistics (median, 25 and 75 percentiles). For readability, the *y*-axis scale is different between Mesagne and Avetrana. ns: non-significant (Kruskal–Wallis *p* > 0.1); a and b indicate different groups (Dunn adjusted *p* < 0.1).

The *tNDVI* tended to decrease with more severe symptoms and bacterial load. In Mesagne, *tNDVI* differentiation by the disease severity class became visually consistent, but rarely statistically significant, from September 2021, with classes 2 and 3 (Figure 4a). From this date, the mean decrease in the *tNDVI* between adjacent symptom severity classes was −35%. The differentiation by symptom classes did not hold in July 2022 between classes 3 and 4. The delayed *tNDVI* response to symptoms could be attributed to the fact that the first wilted branches due to OQDS appeared in the upper canopy and were eventually not captured by TreeTalkers in this initial phase. The *tNDVI* could not clearly differentiate the *Xfp* cell concentration classes in Mesagne (Figure 4c), either statistically or visually, except in May 2021 and April 2022. This is partly due to the poor sample size distribution between the bacterial cell concentration classes, but also the rapid fluctuations of the bacterial load observed in Mesagne in the summer of 2021. In Avetrana, non-symptomatic trees (class 0) tended to have a higher *tNDVI* than symptomatic trees of classes 1 and 2, although the difference was rarely significant (Figure 4b). The mean decrease in the *tNDVI* between adjacent symptom severity classes was −91%. Up to December 2021, only one tree was infected, and most of the symptoms were drought-induced. The detection of infected trees of class 1 from healthy trees of class 0 became statistically significant in June 2022 (Figure 4d). From December 2021, the mean decrease in the *tNDVI* between adjacent *Xfp* concentration classes was −94%. The applied treatments were not a major driver of the *tNDVI*, and the emerging patterns were neither significant nor consistent between the two sites.

The detection capability of the individual spectral bands was assessed at the survey dates (see Supplementary Figure S4 for an example of December 2021). In general, diseased trees were characterized by increased transmittance in the visible region of the radiation spectrum. The band centered on 450 nm and the interval 600–650 nm were the most sensitive to disease severity and *Xfp* cell concentration.

## 3. Discussion

### 3.1. Short-Term Efficacy of the Thymol-Extract Therapies

A novel, natural therapy based on thymol extract solution was tested on two olive groves in the *Xfp*-infected zone of Brindisi and Taranto to investigate the short-term effects (1.5 years of application). The application protocol, with foliar spraying every month from early spring to late autumn, was conceived to be easily replicable in local olive orchards, with no hazards to human and environmental health. In previous in vitro and in pot experiments [29,30], a thymol extract solution proved to be the most effective antibacterial agent among the tested natural extracts. Following the promising results achieved in biomedical applications, the study of nanostructured materials has gained increasing attention in recent years in the agricultural sector. Numerous advantages could be derived from these encapsulated matrices: (1) prolonged and controlled release of agrochemical products; (2) greater efficiency compared to traditional pesticides; (3) reduction in the number and frequency of applied phytosanitary products; (4) greater and more uniform crop protection; (5) reduction in environmental pollution; and (6) better management of resistance phenomena in pathogens [30,31]. Considering these numerous advantages, we tested the thymol solution alone and encapsulated in cellulose nanoparticles. In the preventive trial of Avetrana, a modest reduction in the bacterial cell load was observed when the thymol-based therapies were applied, but the treatment effect was not statistically significant, although it tended to increase over time after one year from its first application (Figure 1e). No effects were recorded in the curative trial of Mesagne (Figure 1f). The encapsulated therapy showed the highest sap flux density reduction in the diseased trees, though the effect was not always consistent over time and between the sites and was not statistically significant, with one exception in Avetrana (Figure 3c,d). The short-term application of the thymol-based treatments was a clear limitation of the results, as there was evidence of a cumulative effect in decreasing the *Xfp* cell concentration in the preventive trial of Avetrana starting from the spring of 2022. Since we did not observe any bacterial load alleviation in the curative trial of Mesagne, we cannot conclude whether the treatment is ineffective when applied to already diseased trees or even longer lag times are needed in diseased trees before its effects unfold. While we cannot conclude that the application frequency was insufficient, the data do not support the hypotheses of field degradation of the thymol products, since the liquid solution and the encapsulated therapy had similar effects. While the thymol-based solution is rapidly available when sprayed on the leaves due to its lipophilicity [14], limited persistence on the leaves of the essential oils was reported due to evaporation and photodegradation [32]. The porous structure and hydrophilic nature of the cellulose nanoparticles can prevent environmental losses and control the solution release by the diffusion and gradual biodegradation of the carrier itself. Furthermore, cellulose nanoparticles can facilitate passive diffusion across the cuticular waxes and stomatal entries thanks to their enhanced adhesion to and retention in the foliar cuticle [33]. Therefore, even if no clear conclusions can be drawn, future investigation of the cellulose nanostructured encapsulation is warranted.

Our results indicate that *Xfp* cell proliferation can be suppressed at high temperatures, and bacterial cell death can be induced when temperatures are extremely high. TreeTalkers revealed that the sapwood, which is in thermal continuity with the xylem where the *Xfp* cells proliferate, can have temperatures a few degrees higher than the ambient air during heatwaves. The *Xfp* cell load reduction observed in Mesagne in the summer of 2021 (Figure 1c) was associated with perceived sapwood temperatures of c.a. 44 °C, 3 °C higher than the corresponding 2 m air temperatures (Figure 2a). In the summer of 2022, *Xfp* concentrations remained stable in the two sites and were associated with sapwood temperatures of c.a 39 °C and 40 °C in Mesagne and Avetrana, respectively, 1 °C and 2 °C higher than the air temperatures. These observations agree with recent evidence that *Xfp* cells are vulnerable to very high temperatures. In [15], temperatures above 36 °C from July to August reduced the bacterial concentration in the olive twigs; in [34], incubation periods of 7 days from 36 °C to 40 °C killed the bacterial cells.

### 3.2. Tree’s Physiological Response to Xfp

The *J* values obtained in this study were comparable with those reported by [35] for the development of the transient thermal dissipation method and with sectorial experimental data. For example, ref. [36] reported a maximum sap flux density of 12 L dm^−2^ h^−1^ in well-irrigated olive trees of the cultivar Arbequina, comparable to the highest *J* values reported in this study. Our results show that *J* responded to the disease severity in Mesagne, with a few exceptions at the beginning and end of the trial, although high data variability and poor sample size distribution hindered the statistical significance (Kruskal–Wallis *p* < 0.1) (Figure 3). One explanation for *J* not responding to *Xfp* cell concentration rate in Mesagne is that the bacterial load fluctuated at this site, particularly in the summer of 2021. A different explanation can be given for *J* not responding to the symptoms in Avetrana: here, the symptoms were mainly drought-induced until December 2021 and were milder than in Mesagne after the grove’s first infection in December 2021. In addition, in Avetrana, there was an indication that *J* could be an early signal of *Xfp* infection (Figure 3), although the few data available warrant caution. These findings globally confirm the tight connection between the plant vascular system and the *Xfp* disease and underscore the promising exploitation of the plant physiological parameters for monitoring the disease, as suggested by [27]. The increase in *J* can be explained as a water redistribution effect. *Xfp* infection has been associated with a significant reduction in hydraulic conductivity in affected hosts [6,37]. Also, it was demonstrated that the xylem conduits in trees can have a large overcapacity [38]: when part of the conducting area loses functionality, trees can adapt to fulfill their water demand by re-routing the sap towards the functioning conduits, which then experience an increased sap-flux density. The highest increase in sap flux density occurs close to the functional disruption [38]. In this work, the observed increase in *J* can be attributed to xylem conductivity disruption in the trunk at breast height, close to TreeTalkers’ measurement point. The functional disruption can be caused by biofilm clogging if the bacterial cells colonize the trunk xylem or by tyloses, gums, and pectin gels induced by the plant’s defenses, particularly at the early phases [22,23]. Furthermore, the increase in *J* with the disease severity observed in Mesagne might be attributed to the cumulative loss of xylem functionality due to cavitation. Indeed, the susceptible cultivars, like Cellina di Nardò, feature fewer and larger xylem vessels than the resistant cultivars, making them more prone to cavitation [24]. Xylem embolism, a relevant threat in the Mediterranean dry environments where olive cultivation is practiced, is exacerbated by the *Xfp* infection in the susceptible hosts. Host colonization proceeds between adjacent vessels through pit membrane degradation and consequent xylem vessel embolism [23]. In this study, we observed that the sap flux density in Mesagne was generally higher than in Avetrana during the hot and dry summer seasons when xylem embolism is more likely to occur (Figure 2). When the soil water availability was restored, embolized vessels were eventually refilled, and the sap flux density between the two sites returned to a similar level. *J* showed some responsiveness to the thymol therapies in Mesagne, although the signal was confused in Avetrana. This effect should be attributed to the therapy capability of restoring the hydraulic conductivity of the xylem thanks to its antibacterial and antibiofilm properties [13,14].

Our study indicates that moderately infected or symptomatic trees did not lose the hydraulic continuum but exhibited well-regulated transpiration fluxes (Figure 3). Non-symptomatic leaves of the infected trees did not report any reduction in water transpiration, indicating a functioning stomatal control (Table 1 and Table 2). Stomatal conductance and transpiration were higher in Mesagne than in Avetrana in the morning, despite the higher bacterial loads. The increased leaf transpiration can also be explained as a water redistribution effect: in symptomatic trees with unaffected root systems, the same water flow is directed through the (reduced) healthy part of the crown, whose leaves thus experience a surplus of the water supply. This increase is likely to occur in the night and early morning when the soil water pool is replenished. The leaves collected in Mesagne had higher *J_max_* than in Avetrana. Considering the relationship with *N_area_*, this increase in *J_max_* could be attributed to the greater, but not significant, thickness and chlorophyll content of the leaves in Mesagne. Except for heavily damaged trees, we did not observe any reduction in water transpiration or stomatal conductance, as reported by [27] in pot experiments. This suggests that leaf functioning in the olive crowns can be very heterogeneous between healthy and symptomatic leaves.

### 3.3. Xfp Monitoring Through Transmitted Radiation

TreeTalker’s transmitted radiation has been used in other studies in the literature to monitor tree phenology [39] and forest management practices [40]. The *NDVI* is regarded as a canopy greenness index and is typically calculated from the visible and near-infrared light reflected by the vegetation. In this study, the *tNDVI* was based on the transmitted radiation instead of the reflected radiation, which exhibits similar curves (i.e., more absorbance in the red region and less in the near-infrared region), although it differs in intensity [41,42,43,44,45]. In Avetrana, the *tNDVI* differentiated, but rarely significantly, infected from non-infected trees at early stages by lowering the index values (Figure 4). In Mesagne, the differentiation of the olive trees according to the *Xfp* cell concentration classes was confounded by both the poor sample size distribution between the classes and the bacterial cell load fluctuations. It can be hypothesized that the fast bacterial cell mortality did not cause simultaneous changes in the leaf pigment status and spectral properties, which were likely driven by the experienced drought. The distinction between the disease severity classes was also delayed in Mesagne (Figure 4). A possible explanation is that the OQDS disease affects the upper branches first, which might not be visible to TreeTalkers. Indeed, in Avetrana, where the initial symptoms were not caused by *Xfp* infection but were drought-induced and distributed in the whole canopy, TreeTalkers differentiated symptomatic from non-symptomatic trees. The scaling procedure, applied to compare the data between sensors, in Mesagne took as a reference the first week of acquisition in October 2020, thus spectrally assimilating healthy and infected trees. To rule out any influence of the scaling on the observed delay, the unscaled data were also analyzed: while being more confounded, the unscaled data displayed the same delay. The transmitted radiation did not consistently identify the effect of the applied thymol-based therapies during the short-term trials. This result agrees with [13], in which recovery of *Xfp*-infected trees became detectable two years after the first treatment application. TreeTalkers’ sensors mainly exploited alterations in the visible spectra, notably in the blue (450 nm) and orange bands (600–650 nm). The significance of the blue region for *Xfp* disease detection was reported by [16] and associated with chlorophyll degradation processes [46]. The findings of this work suggest that the hydraulic processes might respond before the canopy spectral properties as monitored through the transmitted radiation. While TreeTalkers’ spectrometers have the potential to simply and continuously monitor the early stages of *Xfp* infection, this needs to be validated in future studies. One key advantage compared to remote sensing is that TreeTalker’s signals are not affected by the understory. The authors of [17] demonstrated the use of high spatial resolution (2 m) satellite imagery to assess the recovery of olive trees aged from 40 to 100 years. Motivated by this experience, we tested 3 m resolution RGB and NIR bands from Planet to detect disease progression in the 20/25-year-old trees of the present trials. After clipping the individual tree crowns, the presence of different scene components in the pixels restricted the use of the reflectance information in the summer season, when the background is stable, bare soil. Nevertheless, even in the summer, tree differentiation according to disease severity and *Xfp* cell load was severely hampered (Supplementary Figure S5), making TreeTalkers more suitable than satellite imagery in this application.

### 3.4. Limitations and Perspectives for Improvement

The integration of advanced proximal sensing technologies has emerged as an interesting approach in plant pathology for the early detection and precise monitoring of diseases [47]. These non-invasive tools facilitate the real-time assessment of plant physiological responses to biotic stressors, guiding timely interventions and informed disease management strategies. The most frequent tools reported in the literature encompass thermal, fluorescence, and spectroscopic sensors [48]. Here, we applied, for the first time, in the context of *Xfp* epidemics, sensors directly implanted in the tree to monitor the sap flux density and transmitted radiation. While this study provides insights into the use of thymol-based treatments and sensor-based monitoring in olive trees affected by *Xfp*, several aspects warrant further investigation. The performance of TreeTalker’s sensors in differentiating the *tNDVI* between infected from uninfected trees was suboptimal in the early stages of infection, highlighting the need to refine detection algorithms and enhance sensitivity or to couple this monitoring technique with other methods, particularly for early-stage diagnosis. Nonetheless, their non-invasive, continuous data acquisition remains a promising feature for integrative disease monitoring.

Furthermore, this study focused primarily on phenotypic responses, without integrating molecular or cytological analyses that could clarify the plant’s defense mechanisms or the mode of action of thymol. Future research should adopt a multidisciplinary approach, combining physiological, biochemical, and molecular tools to gain a more comprehensive understanding of the host response and to optimize treatment protocols. These improvements would strengthen the robustness and applicability of nature-based solutions in the sustainable management of *Xfp* in olive agroecosystems.

Finally, one of the main findings shows that the therapeutic effect of thymol was limited, with no statistically significant reduction in disease severity or bacterial load observed over the two-year period. This outcome may be influenced by the short duration of the trial and the limited number of treated and monitored trees (n = 40), which constrain the statistical power and temporal resolution of the results. Expanding the experimental scale and extending the monitoring over multiple seasons would further allow us to capture potential long-term effects and seasonal variability in host–pathogen dynamics.

## 4. Materials and Methods

### 4.1. Study Sites

**Figure 5:**
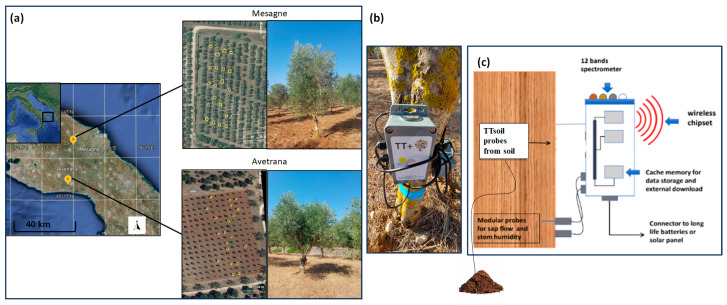
(**a**) Location of the two study sites in the infected zone of Brindisi and Taranto provinces, Apulia region; the olive trees included in the trials are demarcated by contoured crowns. (**b**) Olive tree equipped with TreeTalker. (**c**) Schematic representation of the TreeTalkers’ components.

The two field trials were carried out in the epidemic areas of progression of the *Xfp* disease in Brindisi and Taranto provinces (Apulia), Southern Italy (Figure 5a). One grove (40.576° N, 17.749° E) was close to Mesagne on the Adriatic side of the Brindisi province; the other grove (40.341° N, 17.707° E) was close to Avetrana on the Ionic side of the Taranto province. The two sites have similar Mediterranean climate conditions, with a mean annual temperature of 16.3 °C and a mean annual precipitation of 516 mm year^−1^. The mean summer temperature is 30–33 °C in both sites, but occasionally it can exceed 40 °C. The two privately owned groves were planted with the susceptible cultivar Cellina di Nardò. The olive trees were 20 and 25 years old in Mesagne and Avetrana, respectively, with mean diameters at breast height of 11.7 cm and 19.3 cm. The groves were equipped with drip irrigation systems for summer emergencies, but during the trials, the olive trees were not irrigated. During the trials, the trees were not fertilized or harvested, and the olive fruits fell naturally on the ground.

### 4.2. Field Experiment

At the beginning of the trials in October 2020, n. 20 olive trees per grove (n. 40 trees total) were selected. For each tree, *Xfp* cell concentration was evaluated by real-time quantitative polymerase chain reaction (qPCR) according to the protocol described by [49] using twigs and leaves collected from the four quadrants of the trees according to EPPO protocols [50]. At the same time, plants were scored for *Xfp* symptoms of leaves and shoots on a scale of five levels. Trees with 100% green foliage were assigned to class 0. The subsequent classes were defined as the proportion of the canopy affected by the typical *Xfp* symptoms: 1–3 indicated trees displaying symptoms in 25%, 50%, and 75% of the total branches, and 4 corresponded to trees with a prevalence of dead branches. In Mesagne, at the start of the trial, all selected plants showed *Xfp* symptoms (severity classes ranged between 1 and 2), and one-third of the plants loaded the bacterium; therefore, the selected trees were assigned to each treatment for not having statistically significant differences among treated and untreated plants. In Avetrana, since none of the trees were infected or symptomatic at the inception, the selection was performed using a randomized block design (see Supplementary Figures S6 and S7 for the experimental design in Mesagne and Avetrana, respectively). Three treatments were applied to the selected trees: untreated control (*ctr*) in n. 6 trees, foliar spraying of 3% thymol extract solution (*thym)* supplied by Licofarma S.r.L., Galatina, Italy, to n. 7 trees, and foliar spraying of the same 3% thymol extract solution encapsulated in cellulose nanoparticles (*thym + nano*) supplied by Spagro S.r.L., Barletta, Italy, to n. 7 trees. Thymol extract was selected among other natural products for having the strongest antibacterial effect [29,30]. The application of *thym* started in May 2021, and for the first three months, it was extended to the *thym + nano* trees owing to nanoparticle unavailability. From August 2021, *thym* and *thym + nano* treatments were regularly applied once per month with suspension during winter until October 2022. Although the delayed start of the *thym + nano* treatment may introduce a potential temporal bias when comparing the two treatments, its impact is likely limited, as both groups were subjected to comparable conditions during the main period of treatment and monitoring.

Site surveys and sampling were carried out every two months, except during the winter dormancy breaks. In analogy with the symptoms, the *Xfp* cell concentrations were classified according to a 0–3 rating scale: class 0 corresponded to healthy trees, class 1 to low infection trees (bacterial cell load ≤ 3.3 E + 03 CFU mL^−1^), class 2 to intermediate infection trees (bacterial cell load ≤ 4.4 E + 05 CFU mL^−1^), and class 3 to high infection trees (bacterial cell load > 4.4 E + 05 CFU mL^−1^). Within each site, the genetic uniformity of the three (belonging to the same cultivar), their similar age and size, and the homogenous soil characteristics ensured low variability in the leaf physiological traits among trees. Four olive trees per site were therefore considered an adequate sample size for leaf physiology monitoring [51,52]. Leaf physiological parameters were determined from 9 June to 10 June 2021.

The apparent maximum rate of carboxylation by Rubisco (*Vc_max_*) and the ribulose diphosphate maximum regeneration rate (*J_max_*) were calculated based on photosynthesis-CO_2_ response curves (*A-C_i_* curves) [53], using a LI-6400 portable photosynthesis system (LI-COR Instruments, Lincoln, NE, USA). Non-symptomatic twigs from the four quadrants of the trees were excised the day before the measurement, but were not tested for *Xfp* cell concentration. Cutting was performed underwater to prevent xylem cavitation, and the excised twigs were kept with the bases immersed in water until measurement. This procedure provides, even during dry periods, full hydration of the leaves and, therefore, adequate stomatal conductance necessary to obtain a correct photosynthetic capacity estimate [54]. In the leaf chamber, quantum flux density was set to 1500 μmol photons m^−2^ s^−1^ and air temperature to 25 °C. However, because of the high temperatures recorded during the measurement days, the temperature control unit of the LI-6400 failed to maintain 25 °C inside the leaf chamber, and leaf temperature ranged between 25 and 30 °C. *Vc_max_* and *J_max_* values were therefore corrected to obtain the parameter values at 25 °C according to [55,56]. After measurements, leaves were collected, scanned to determine leaf area, and then dried at 70 °C to obtain the dry weight. Leaf mass per area (*LMA*, g m^−2^) was calculated as the ratio of leaf dry weight to leaf area. The leaf samples were then ground, and the carbon (*C*, %) and nitrogen (*N*, %) contents were determined using an elemental analyzer (Carlo Erba model 1108EA, Milan, Italy). On the same days of June 2021, stomatal conductance, leaf transpiration, and leaf fluorescence under actual environmental conditions were measured on attached leaves using a LI-600 Porometer/Fluorometer (Li-COR Instruments, Lincoln, NE, USA). For each tree, 48 leaves were measured with the LI-600 by maintaining the original orientation of the leaves. The leaves selected for the measurement were distributed in the four quadrants and on both the higher and lower parts of the canopy. In addition, both previous- and current-year leaves were measured. This set of measurements was repeated before and after noon for each tree.

### 4.3. TreeTalker Sensors

TreeTalkers are cheap, easily deployable, and maintainable systems for continuous plant monitoring. They consist of a microcontroller with an ATMega 328 processor chip operating the sensors. The microcontroller is placed in a case fastened northward on the tree trunk at breast height (Figure 5b). At the start of the trials in October 2020, the selected trees were equipped with one TreeTalker each (n. 20 sensors per site, n. 40 sensors total). Data acquisition from the TreeTalker’s sensors was set at an hourly frequency and lasted until July 2022. The solar panel-supported Li-ion batteries powering the TreeTalkers were serviced monthly. The data were stored in internal flash memory devices and transmitted via a long-range (LoRa) connection to a master node (the cloud) placed close to the TreeTalkers, which communicated via GSP to a central server [19]. A schematic description of the TreeTalkers is shown (Figure 5c), while more details can be found in the handbook (Nature 4.0 Manual, 2023), available online: https://www.nature4.org/it/treetalkercyber (accessed on 20 April 2025).

Sap flux density, or sap flow velocity, is measured and validated by the manufacturer according to the thermal dissipation method [57], which accounts for the temperature difference between a heated needle inserted in the sapwood and the sapwood some distance below the needle. The temperature difference increases when the sap flux density decreases, reaching a maximum when the sap flow is zero. The modification proposed by [58], which consists of needle heating and cooling cycles, is also available to account for natural temperature gradients. Considering the hot and dry environment of the two sites, the non-continuous heating system was chosen, with cycles of 10 min of heating followed by 50 min of cooling. The two temperature needle probes (±0.1 °C) were implanted in the sapwood 10 cm vertically apart and connected to the TreeTalker case.

Two commercial spectrometers, mounted on the top of the TreeTalker case, measure the light transmitted through the canopy with a field of view of 40°, with one covering the visible electromagnetic region with bands centered at 450, 500, 550, 570, 600, and 650 nm (40 nm bandwidth) and the other operating in the infrared region with bands centered at 610, 680, 730, 760, 810, and 860 nm (20 nm bandwidth) [59]. Due to the absence of a light diffuse filter, only the data acquired after 9:00 AM were retained [39].

TreeTalkers measure air temperature in the under-canopy microclimate. Other climatic data, such as 2 m air temperature, solar radiation, total precipitation, and relative humidity, were retrieved from meteorological stations close to the study sites. In particular, two meteo stations at 6 km from Mesagne in different directions were selected and averaged (OPU34 and OPU35 according to ARIF’s nomenclature), and one meteo station at 8.5 km from Avetrana was selected (OPU44). The microclimatic variations between the meteo stations and the study sites were considered negligible. Vapor pressure deficit (*VPD*) was estimated from the 2 m temperature and relative humidity using the R package REddyProc [60]. Soil humidity and temperature were measured at an hourly frequency with TTsoil (Nature 4.0 Manual, 2023), available online: https://www.nature4.org/it/soil (accessed on 20 April 2025). TTsoil is an extension of the TreeTalker system, consisting of a capacitive probe with a thermistor buried in the soil and rooted to the sensor case. Two TTsoils per site (n. 4 total) were installed, each featuring two probes deployed at a depth of 20 cm on the opposite sides of the tree to avoid plowing disturbances. Undisturbed soil samples were collected close to each probe for laboratory determination of soil bulk density and water retention curves, which were used to calculate soil volumetric water content and water potential, respectively. For each site, a second undisturbed soil sample was collected to develop site-specific calibration curves for gravimetric soil water content as a function of the measured soil temperature and frequency.

### 4.4. TreeTalker Data Processing

TreeTalker’s data were retrieved from the central server, converted from digital numbers to physical quantities, and cleaned from duplicated and unfeasible values using the default settings of the R package ttprocessing [61]. Then, the following semi-automatic pipeline for additional data curation was applied. Data were grouped seasonally (September–November 2020–2021, December–February 2020–2021, March–May 2021–2022, and June–August 2021–2022), and spikes were removed following [62]. Scattered and fluctuating signals were manually removed (see examples in Supplementary Figure S8 for replicability). Unintended spectrometer rotations, which occurred for some sensors in December 2021 due to a windstorm, were fixed by aligning the signals (see an example in Supplementary Figure S8). Sensors presenting anomalous battery voltage trends (Supplementary Figure S8) or poor correlations with the other sensors were flagged for removal testing. For correlation, the Pearson coefficients calculated for all sensor pairs were averaged, and the sensor–season pairs with z-scores higher than 2.5 were flagged. As for the sap flux density, sensors with poor correlation with air temperature and *VPD* (z-scores > 2.5 for both) were also flagged for removal testing. The seasonal groups were re-aggregated, and each sensor’s data were interpolated with a local polynomial regression using the loess function with frac = 0.2 of the python package statsmodels [63]. Data beyond 3.5 standard deviations from the interpolating function were removed. As for the sap flux density, all the hourly data in a day were dropped when more than six observations per day were missing. For the transmitted radiation, all the hourly data in a day were dismissed if fewer than four observations per day were available. Finally, a smoothing function, based on local polynomial regression with frac = 0.0005, was applied to preserve the sub-diel patterns for the sap flux density.

Then, the data were transformed into the final variables of interest and scaled with reference values to compare the time trends between the sensors. The sap flux density *J* (l dm^−2^ h^−1^) was calculated according to [35]

(1) $$J=12.95(\frac{{dT}_{min}}{dT}-1)$$

(2) $$dT=∆T_{h}-∆T_{c}$$

where *dT_min_* (°C) is the transient signal at zero-flow condition, and *dT* is the transient signal (°C) given by the difference between the differential temperature after the heating period *AT_h_* (°C) and the differential temperature after the cooling period *AT_c_* (°C). As in [64], we did not assume that *dT_min_* occurs every night, i.e., the sap flux density goes to zero every night. Instead, we used a single *dT_min_* as a reference value for each sensor, which was taken as the minimum sap flux density recorded in the winter of 2020. The assumption of not forcing *J* to zero every night (or every few days) can be more reliable in hot and dry environments when water replenishment occurs overnight. This assumption emphasized the seasonal variations in *J* with improved interpretability, but it did not alter the general patterns of *J* (Supplementary Figure S9) or *J* responses to the OQDS disease and applied treatments at the survey dates.

The Transmittance-based Normalized Difference Vegetation Index *tNDVI* was used to assess the radiation transmitted through the canopy as [33]. Here, the *tNDVI* was calculated from the digital values acquired by the TreeTalker’s spectrometers without conversion to energy units, according to

(3) $$tNDVI\;=\;\frac{T_{810}+T_{650}}{T_{810}-T_{650}}$$

where *T_810_* and *T_650_* are the digital values of the transmittance bands centered at 810 nm and 650 nm, respectively. The *tNDVI* was scaled by subtracting the mean of the first week of acquisition as the reference value for each sensor. The first week of acquisition was in October 2020 for Mesagne, when half of the olive trees were already infected and mildly symptomatic, and olive fruits had not dropped yet, and in March 2021 for Avetrana due to a cloud fault, when olive trees were healthy and free of fruits. The scaling was critical for sensor intercomparison, as the TreeTalker spectrometers were influenced by features present within the sensor field of view, such as branches that lowered the *tNDVI* regardless of leaf scorching and desiccation. Due to the different scaling windows, site comparison of the *tNDVI* time trends was not possible. *J* was gap-filled when fewer than 6 hourly observations per day were missing (otherwise, hourly data were discarded for the whole day). Random forest models were trained for each sensor–season combination using the python package scikit-learn [65]. Air temperature, radiation, precipitation, soil water content, and sap flux density estimated from a single probe, when available, were used as predictors. Fitted models with a Pearson correlation coefficient lower than 0.7 were discarded and replaced by time linear interpolation models. Daysum *J* (l dm^−2^ d^−1^) and daymean *tNDVI* were finally calculated.

### 4.5. Statistics

To assess the effect of the thymol-based therapies on the symptom severity and *Xfp* cell concentration, the non-parametric Kruskal–Wallis test was performed at the survey dates because the assumption of normality was often not verified. The Cohen’s d test was used in Avetrana to assess the treatment effect sizes on *Xfp* cell concentration at the survey dates. Before testing, the inverse hyperbolic sine transformation (*arcsinh*) of the *Xfp* cell concentration was taken to improve the data distribution and readability. However, the statistical tests were not altered by the *arcsinh* transformation of the *Xfp* cell concentration values. To compare *J* between symptom classes, *Xfp* concentration classes, and treatments, three days close to the survey dates, free of rain, and with similar conditions of mean temperature, solar radiation, and *VPD* were selected by k-means clustering with the scikit-learn package. Non-parametric bootstrapping, with 1000 samples drawn with replacement to estimate the mean and confidence interval of temperature, solar radiation, and *VPD*, confirmed the clustering robustness (Supplementary Table S1). The daysum *J* values corresponding to the selected days were averaged before the statistical tests. To compare the *tNDVI* between classes and treatments, 7-day intervals centered on the survey dates were selected; the daymean *tNDVI* values corresponding to the selected interval were averaged before the statistical tests. The removal of the flagged sensor–season pairs was tested: if data uncertainty within groups was reduced, the removal was confirmed. The non-parametric Kruskal–Wallis test was used for *J* and the *tNDVI*, as the assumptions of normality and homogeneity of variance were rarely met. When differences were significant, the group means were compared using the non-parametric Dunn’s post hoc test with Bonferroni correction. Differences between sites in leaf traits were assessed with a t-test after verifying the assumptions of normality and homogeneity of variance with Bartlett’s test. The statistical tests were performed with the python package scipy [66]. The results were statistically significant when *p* < 0.1. Programming codes and figures were made in python v 3.10.

## 5. Conclusions

A novel thymol-based therapy was tested on *Xfp*-diseased trees in the infected zone of Brindisi and Taranto, and the early detection and monitoring capabilities of the TreeTalker sensing system were investigated. In this short-term application, thymol extract therapy with and without cellulose nanoparticle encapsulation had a non-significant but increasing effect in reducing the *Xfp* cell load in the preventive trial, but it had no effect in the curative trial. The cellulose nanostructured therapy was slightly more effective than the solution alone. Sap flux density, as measured by TreeTalkers implanted in the trunk at breast height, was responsive to symptom progression and, less consistently, to therapy application in the curative trial. There was also an indication of early response to *Xfp* infection in the preventive trial, but the data warrant caution. TreeTalkers exploited spectral transmittance through the canopy to detect *Xfp* disease at early stages in the preventive trial and monitor symptom progression, not early symptoms, in the curative trial. The *tNDVI* appeared as an appropriate spectral transmittance index and did not suffer from the understory interference. Although more investigation is needed in longer-term trials with a greater number of trees, the integration of proximity sensing for monitoring sapflow and canopy spectral traits appears to be a promising tool for *Xfp* disease detection and control.

## Figures and Tables

**Table 1:** Leaf trait comparison between Mesagne and Avetrana. Mean values are given with standard deviation in parentheses. *Vc_max_*: apparent maximum rate of carboxylation by Rubisco; *J_max_*: ribulose diphosphate maximum regeneration rate; *LMA*: leaf mass per area; *N*: nitrogen content; *N_area_*: nitrogen content by leaf area unit; *N_area_*: carbon content by leaf area unit. ns: non-significant (*t*-test *p* > 0.1), *: *t*-test *p* ≤ 0.1, ***: *t*-test *p* ≤ 0.001.

Property	Mesagne Mean (sd)	Avetrana Mean (sd)	Significance
*vc_max_* (µmol CO_2_ m^−2^ s^−1^)	69 (17)	71 (15)	ns
*J_max_* (µmol m^−2^ s^−1^)	198 (37)	156 (34)	*
*J_max_/vc_max_*	3 (0.5)	2.2 (0.2)	***
*LMA* (g m^−2^)	230 (47)	216 (40)	ns
*N* (%)	1.6 (0.6)	1.6 (0.2)	ns
*N_area_* (g m^−2^)	3.8 (1.5)	3.6 (0.5)	ns
*C_area_* (g m^−2^)	112 (24.1)	111 (18.9)	ns

**Table 2:** Leaf trait comparison between Mesagne and Avetrana. Mean values are given with standard deviation in parentheses. *gsw*: stomatal conductance; *E*: leaf transpiration. ns: non-significant (*t*-test *p* > 0.1), ***: *t*-test *p* ≤ 0.001.

Property	Mesagne Mean (sd)	Avetrana Mean (sd)	Significance
*gsw* (mol H_2_O m^−2^ s^−1^) before noon	0.164 (0.072)	0.098 (0.039)	***
*gsw* (mol H_2_O m^−2^ s^−1^) after noon	0.098 (0.045)	0.094 (0.046)	ns
*E* (mmol H_2_O m^−2^ s^−1^) before noon	3.26 (1.45)	2.62 (1.11)	***
*E* (mmol H_2_O m^−2^ s^−1^) after noon	2.81 (1.38)	2.98 (1.55)	ns

## Data Availability

The original contributions presented in this study are included in the article/Supplementary Materials. Further inquiries can be directed to the corresponding author.

## Supplementary Materials

The following supporting information can be downloaded at https://www.mdpi.com/article/10.3390/plants14091380/s1, Figure S1: Pearson correlations of *J* with climate variables; Figure S2: *J* in the summer of 2022; Figure S3: Comparison between *J* with leaf transpiration; Figure S4: Sensitivity of TreeTalker’s spectral bands; Figure S5: Remote sensing spectral indexes; Figure S6: Experimental design in Mesagne; Figure S7: Experimental design in Avetrana; Figure S8: Manual cleaning of TreeTalker’s data; Figure S9: *J* patterns with daily scaling; Table S1: Bootstrapping of selected dates.
