# Supplementary material for: Physiological Response of Olive Trees Under Xylella fastidiosa Infection and Thymol Therapy Monitored Through Advanced IoT Sensors
Source: Plants (Basel). 2025 May 2;14(9):1380. doi: 10.3390/plants14091380 (PMC12073793; doi:10.3390/plants14091380)
Supplement: Supplementary file 1 [file plants-14-01380-s001.zip › plants-3584001-supplementary.pdf]

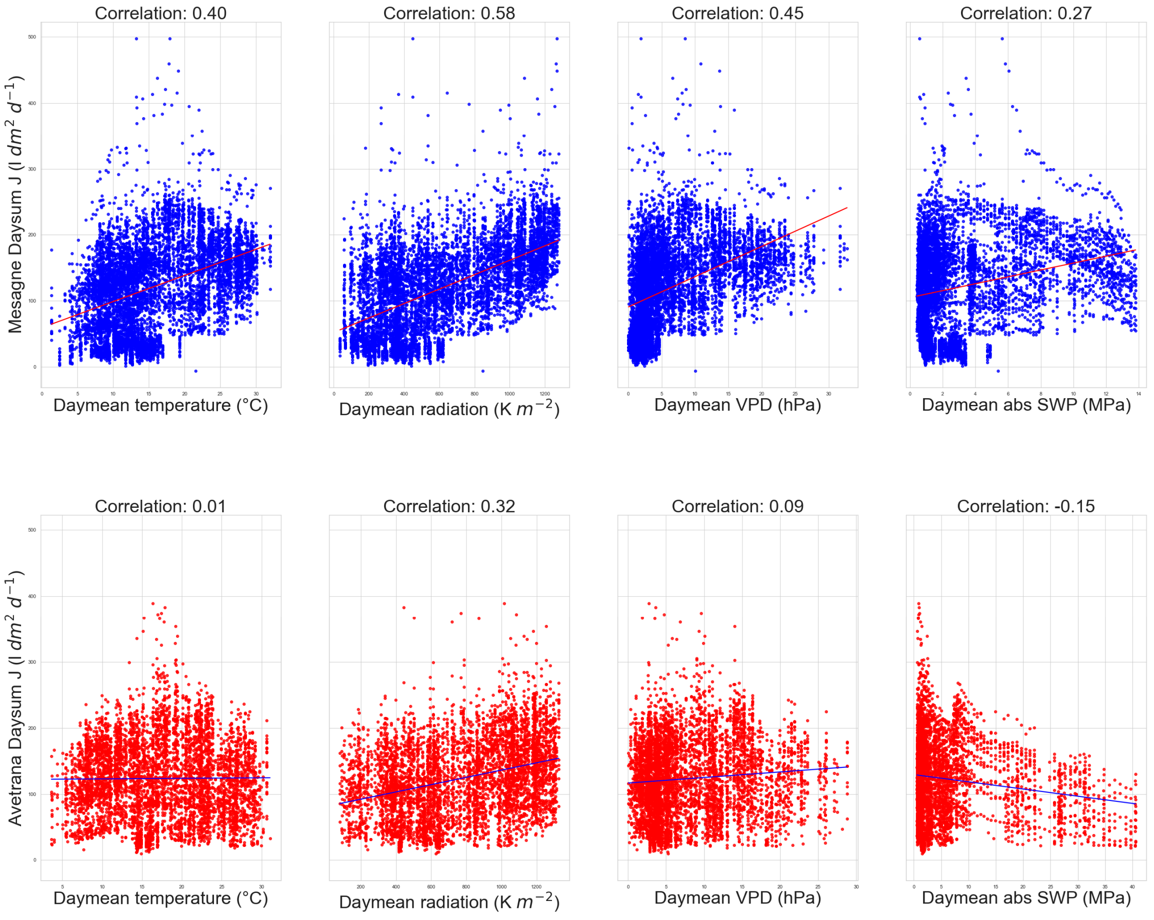

Figure S1: Pearson correlation between daysum *J* and daymean temperature, solar radiation, vapor pressure deficit (*VPD*) and soil water potential (*SWP*) in Mesagne (top figures) and Avetrana (bottom figures).

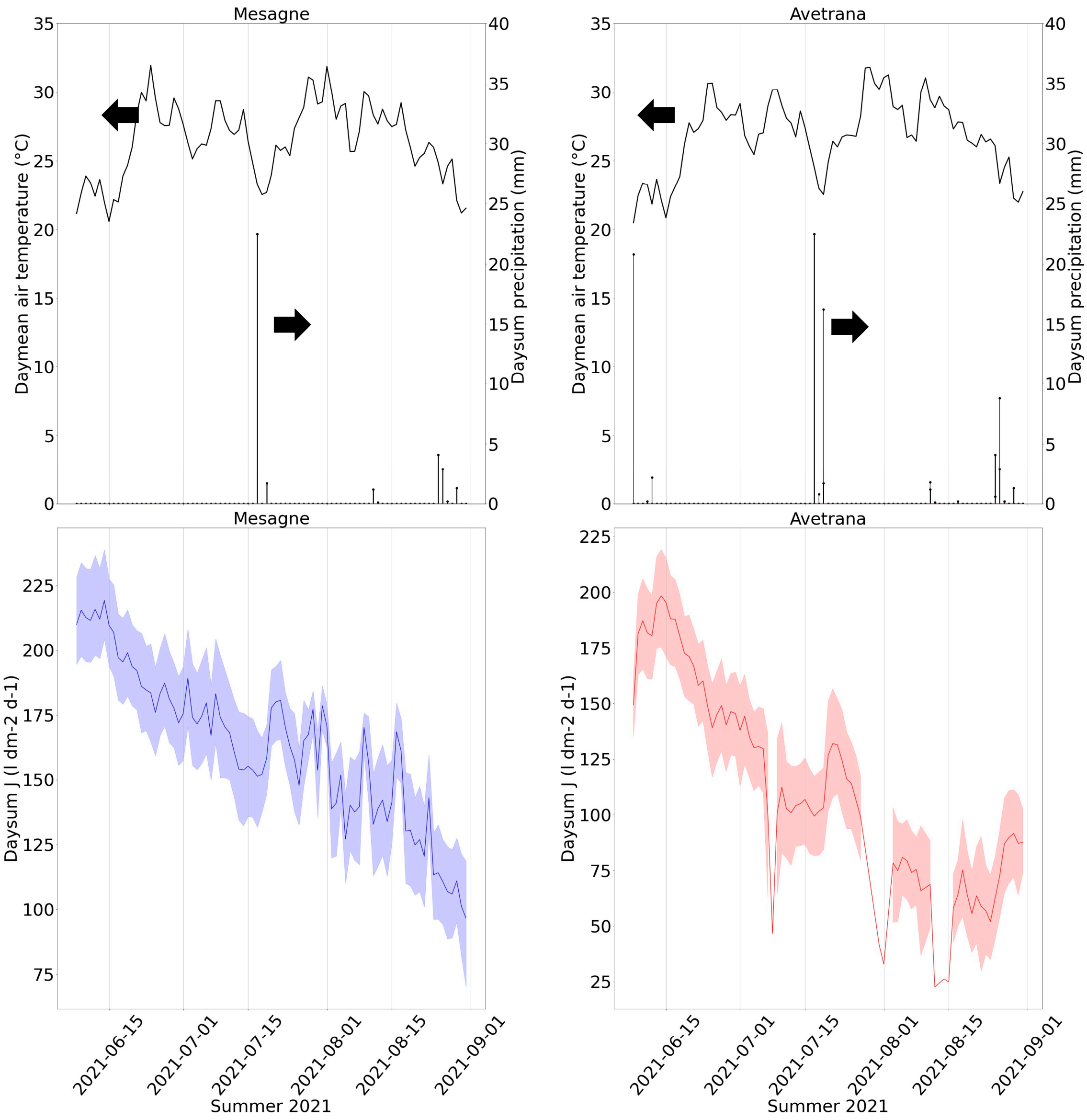

Figure S2: Daymean air temperature (left axis, top figures) and daysum precipitation (right axis, top figures) drive daysum *J* dynamics (bottom figure) during the summer of 2021 in Mesagne and Avetrana sites.

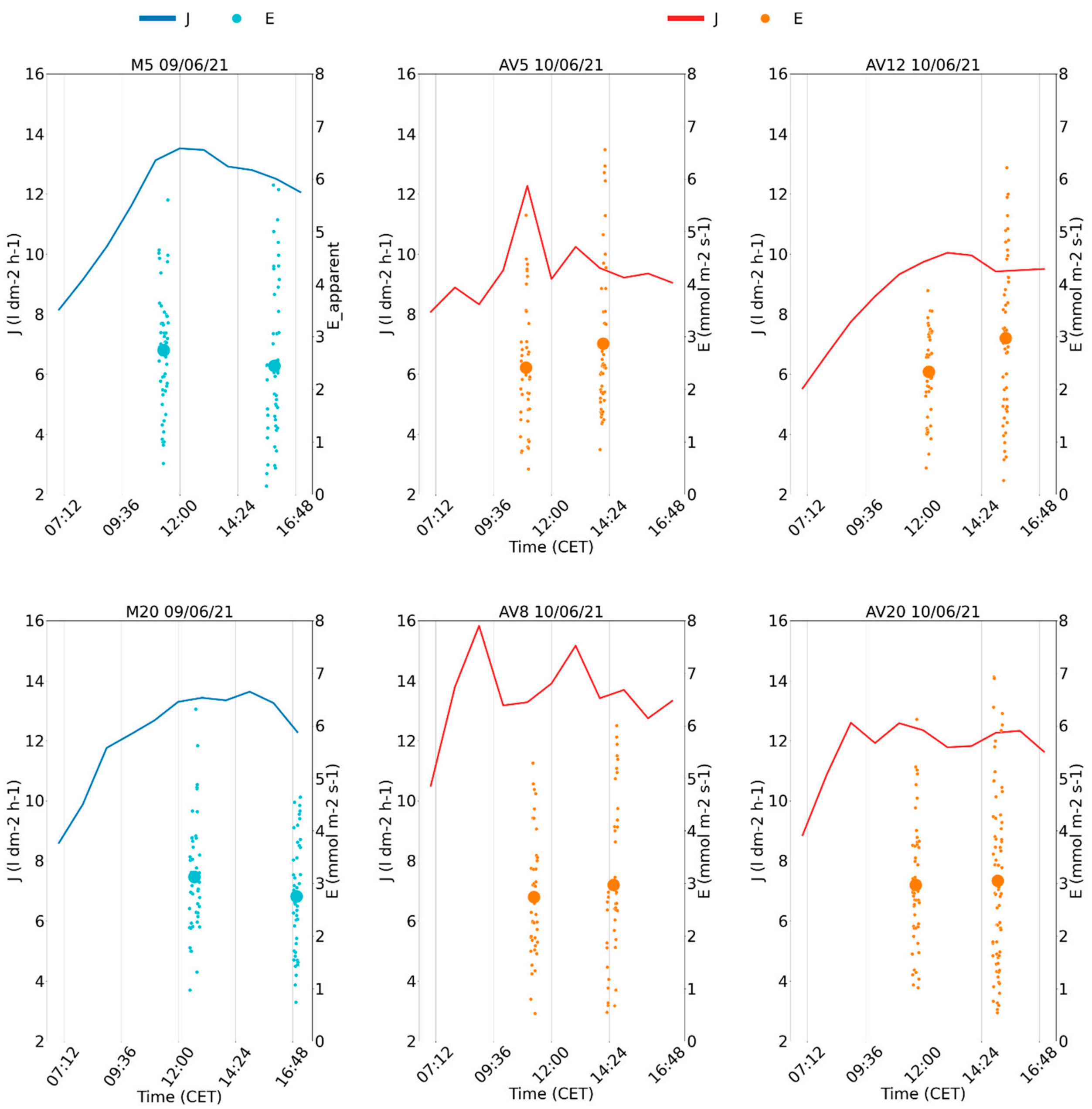

Figure S3: Comparison of hourly *J* with leaf transpiration measurements for the olive trees with tag M5 and M20 in Mesagne, AV5, AV8, AV12 and AV20 in Avetrana on the 9th and 10th of June, 2021.

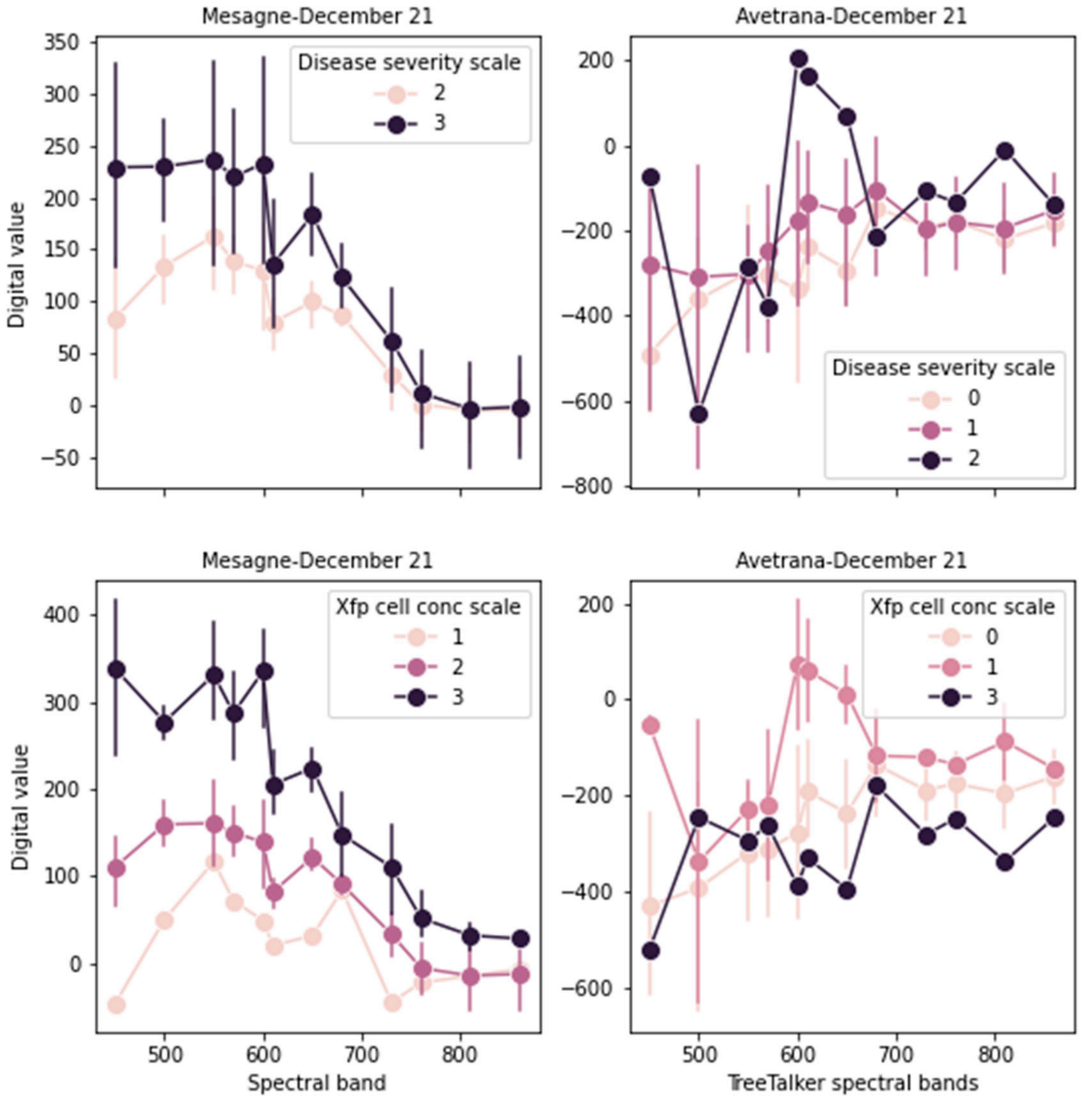

Figure S4: Sensitivity of the TreeTalker's spectral bands to disease severity and *Xfp* cell concentration scales at the December 2021 survey date.

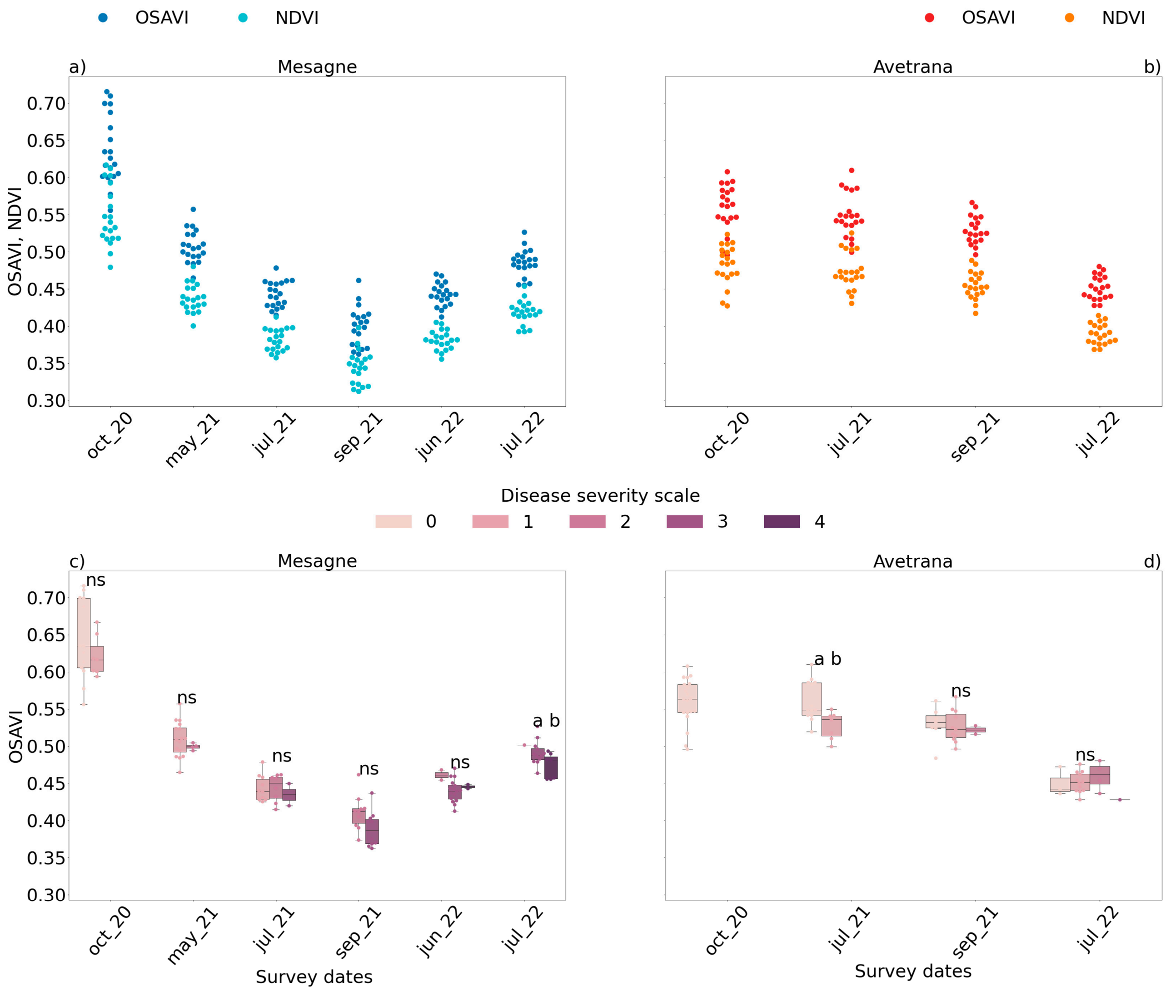

Figure S5: Tree-crown *OSAVI* and *NDVI* at the survey dates derived from PlanetScope high-resolution imagery in Mesagne (a) and Avetrana (b). *OSAVI* at the summer survey dates grouped by symptom severity classes in Mesagne (c) and Avetrana (d).

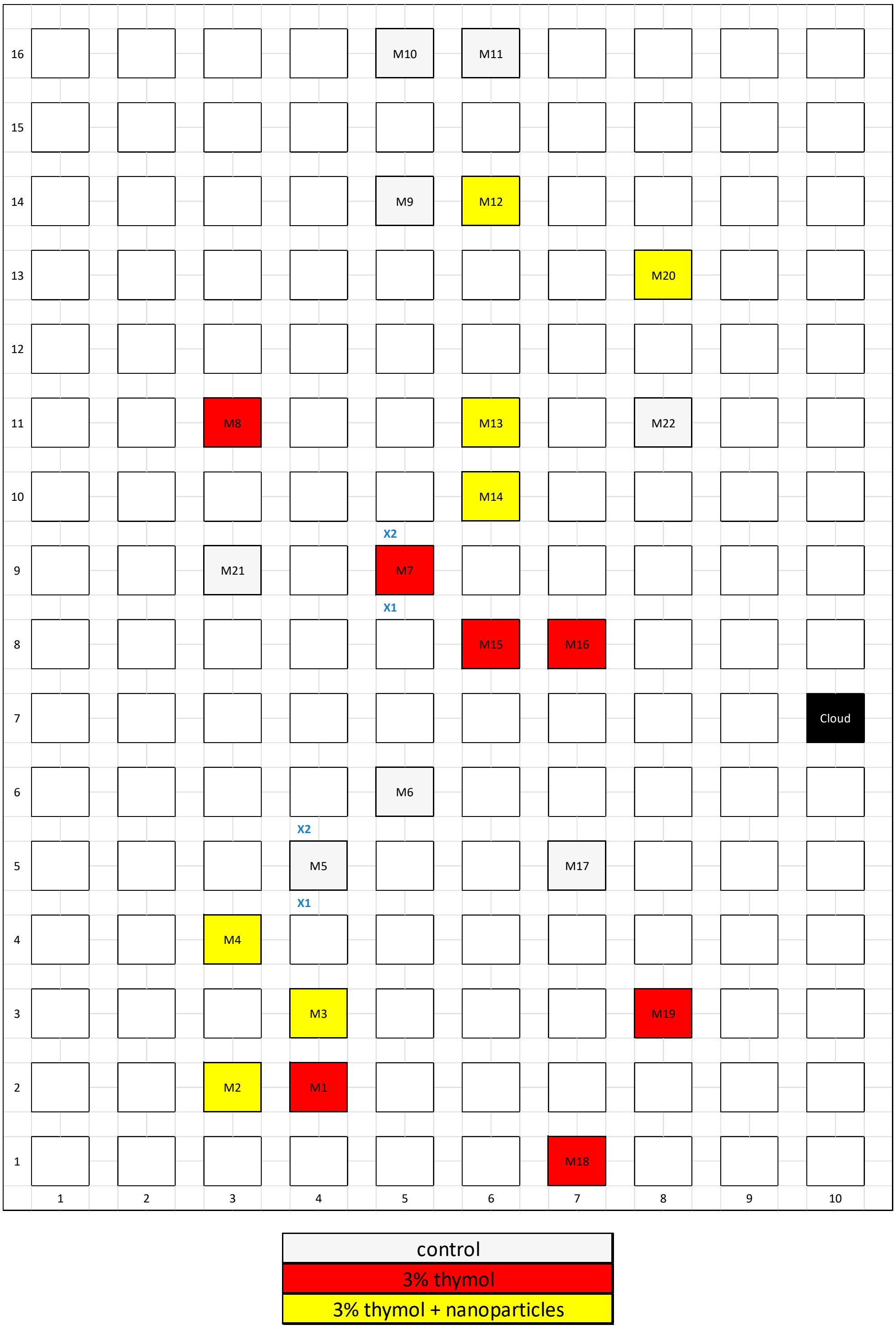

Figure S6: Treatment experimental design in Mesagne. The TreeTalkers are identified by tag, the cloud is marked with a black box and the TTsoils probes are in blue on the opposite sides of the tree.

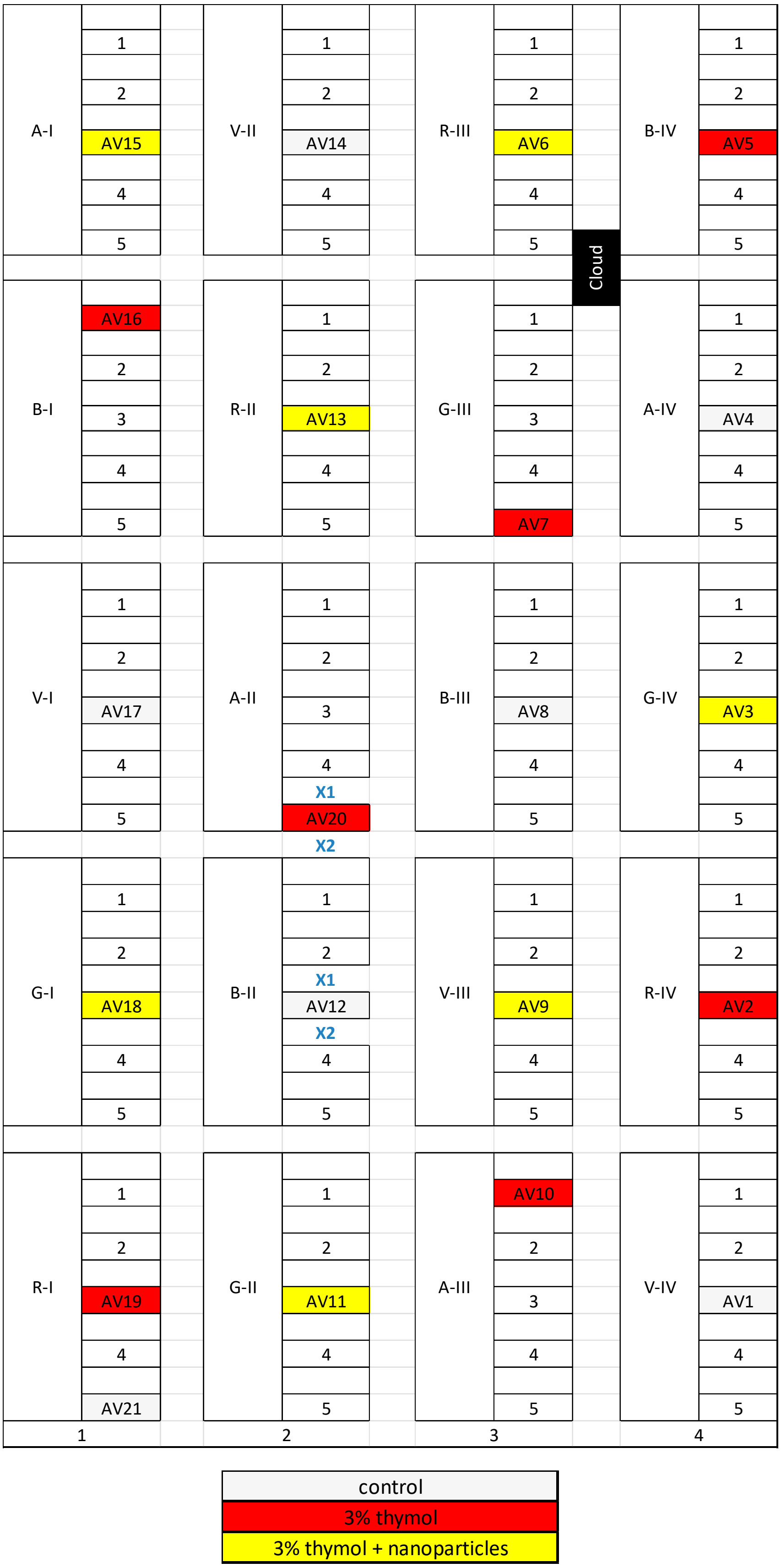

Figure S7: Treatment experimental design in Avetrana. The TreeTalkers are identified by tag, the cloud is marked with a black box and the TTsoils probes are in blue on the opposite sides of the tree.

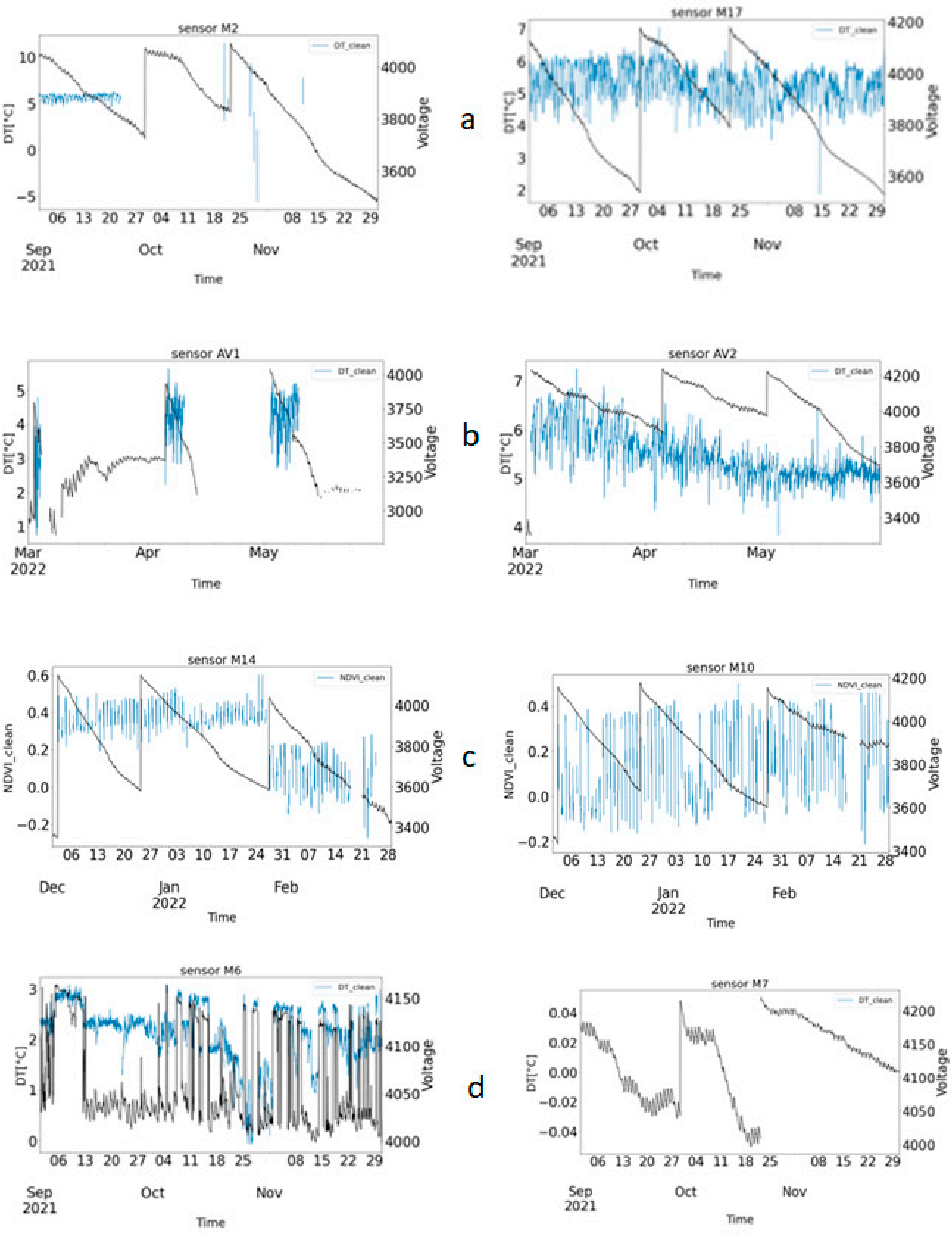

Figure S8: Examples of manual data cleaning in the data curation pipeline: scattered signal (sx) vs reference signal (dx) in blue **(a)**; fluctuating signal (sx) vs reference signal (dx) in blue **(b)**; sensor rotation (sx) vs reference signal (dx) in blue **(c)**; anomalous battery voltage (sx) vs reference trend (dx) in black **(d)**.

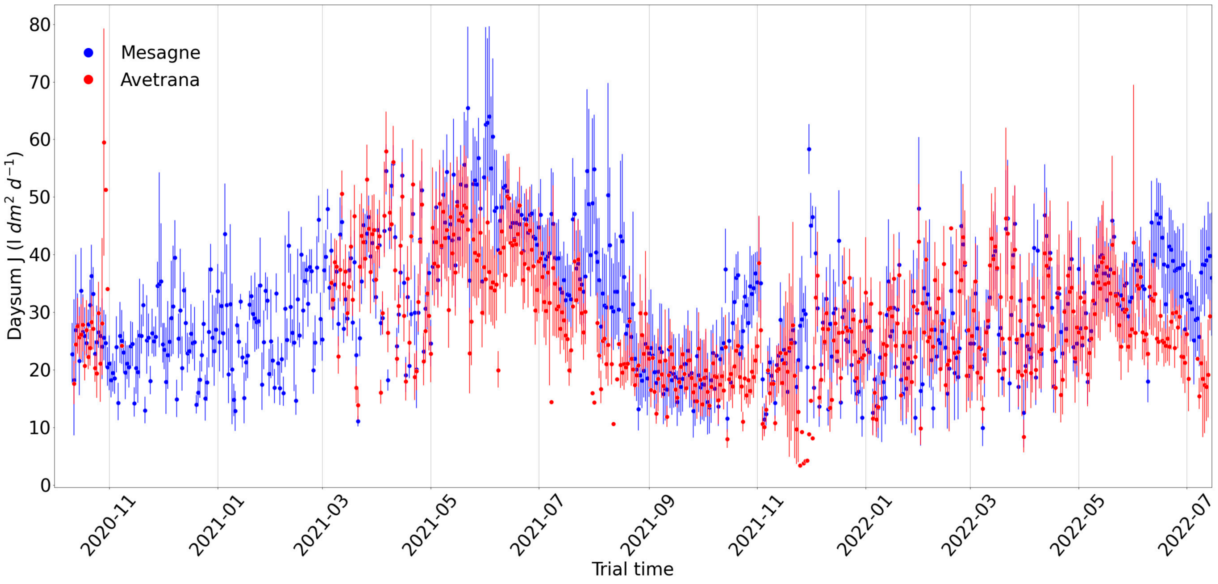

Figure S9: Temporal patterns in Mesagne and Avetrana of daysum sap flux density *(J)* from TreeTalkers when assuming zero flux every night for scaling. TreeTalker's data are shown with the mean and 95% confidence interval.

Visit	Site	Temp (°C) [CI 95%]	Rad (W/m²) [CI 95%]	VPD (hPa) [CI 95%]
**apr_22**	**Avetrana**	11.18 [10.01–12.06]	890.12 [865.67–914.74]	4.76 [4.27–5.46]
	**Mesagne**	11.24 [10.60–11.59]	833.33 [767.28–869.21]	5.46 [4.14–6.33]
**dec_21**	**Avetrana**	8.26 [6.94–9.99]	401.77 [373.70–420.97]	3.41 [3.14–3.62]
	**Mesagne**	12.58 [11.58–13.85]	239.19 [162.80–361.41]	2.17 [1.38–2.89]
**feb_22**	**Avetrana**	12.15 [9.82–14.03]	538.14 [455.02–640.03]	2.43 [1.89–3.07]
	**Mesagne**	11.06 [9.50–12.59]	643.79 [616.20–692.20]	5.33 [4.23–6.00]
**jul_21**	**Avetrana**	25.79 [24.91–26.43]	1167.15 [1065.94–1221.40]	15.10 [10.72–17.67]
	**Mesagne**	25.97 [25.76–26.14]	1176.82 [1155.12–1193.10]	14.85 [14.33–15.81]
**jul_22**	**Avetrana**	29.05 [27.69–30.33]	1064.44 [966.01–1177.78]	23.12 [19.45–26.36]
	**Mesagne**	27.76 [27.64–27.83]	1088.46 [993.21–1208.06]	18.45 [17.59–19.17]
**jun_22**	**Avetrana**	25.22 [24.80–25.63]	1269.27 [1236.50–1290.00]	17.59 [15.80–18.85]
	**Mesagne**	24.53 [23.85–24.98]	1229.27 [1187.49–1252.43]	18.57 [17.16–19.38]
**mar_21**	**Avetrana**	10.25 [9.54–11.01]	591.57 [519.52–636.81]	2.79 [1.81–4.25]
	**Mesagne**	10.33 [8.41–12.08]	715.53 [679.66–735.47]	4.29 [4.17–4.43]
**may_21**	**Avetrana**	18.56 [16.88–19.43]	1039.57 [986.77–1126.59]	9.94 [9.05–10.66]
	**Mesagne**	19.90 [19.04–21.38]	1091.20 [1009.40–1132.31]	9.70 [9.41–10.19]
**oct_20**	**Avetrana**	15.50 [14.75–16.90]	650.66 [643.72–663.96]	5.63 [5.01–6.26]
	**Mesagne**	14.23 [13.27–15.77]	601.89 [589.82–619.26]	4.30 [4.11–4.61]
**sep_21**	**Avetrana**	19.43 [18.60–21.01]	793.08 [723.50–850.62]	10.71 [10.42–10.96]
	**Mesagne**	20.28 [18.48–22.44]	735.92 [672.68–806.26]	8.21 [5.41–9.65]

Table S1: Estimated means and 95% confidence intervals for daily mean temperature (*temp*), solar radiation (*rad*), and vapor pressure deficit (*vpd*) at the Mesagne and Avetrana sites. Estimates were obtained using non-parametric bootstrapping (1000 iterations) for each survey date. Confidence intervals are reported in square brackets next to the means.
